# Combined Effects of Nano-Zinc Oxide and Sesame Oil on Growth Performance, Antioxidant Defense, and Mineral Metabolism in Growing Rabbits

**DOI:** 10.3390/vetsci13090929

**Published:** 2026-09-09

**Authors:** Ahmed A. A. Abdel-Wareth, Mohamed A. Fawaz, Hazem G. M. El-Sayed, Afifi S. Afifi, Abdalla H. H. Ali, Hamdy A. Hassan, Fatma S. O. Elkhateeb, Jayant Lohakare

**Affiliations:** 1 Poultry Center, College of Agriculture, Food and Natural Resources, Prairie View A&M University, Prairie View, TX 77446, USA; jalohakare@pvamu.edu; 2 Department of Animal and Poultry Production, Faculty of Agriculture, Qena University, Qena 83523, Egypt; fawaz@agr.svu.edu.eg (M.A.F.); afifi.salama@agr.svu.edu.eg (A.S.A.); abdalla.ali@agr.svu.edu.eg (A.H.H.A.); hamdy_ahmed@agr.svu.edu.eg (H.A.H.); fatma.saied@agr.svu.edu.eg (F.S.O.E.); 3 Regional Centre for Food and Feed (RCFF), Agricultural Research Centre, Cairo 12619, Egypt; h.elsayed@arc.sci.eg

**Keywords:** nano-zinc oxide, sesame essential oil, heat stress, growth performance, antioxidant defense, mineral metabolism

## Abstract

Heat stress adversely affects productivity and oxidative balance in rabbits. This study evaluated nano-zinc oxide (Nano-ZnO), sesame essential oil (SEO), and their combination in growing rabbits under severe to very severe heat stress (maximum daily THI, 10-day averages, 29.47–30.58). Eighty V-line males (45 days; 1100 ± 55 g) were assigned to basal diet, Nano-ZnO (60 mg/kg), SEO (300 mg/kg), or both for 60 days. Growth, digestibility, carcass traits, serum biochemistry and minerals, antioxidant status, and liver Zn were measured. No mortality occurred. Body weight was higher in all supplemented groups at 85 and 105 days (*p* = 0.001); at 65 days, only Nano-ZnO and SEO groups differed from the control. Overall, 45–105-day gain was higher in all supplemented groups (*p* = 0.001) and feed conversion improved (*p* = 0.009), whereas interval gains differed by additive. Nano-ZnO increased feed intake; SEO reduced intake. SEO, alone or combined, enhanced dry matter and crude protein digestibility (*p* < 0.01), and ether extract digestibility was highest with the combination. Carcass yield was unchanged, but relative liver and kidney weights increased with the combination (*p* ≤ 0.018). Nano-ZnO elevated serum Zn and Mg and liver Zn, whereas Ca decreased; *p* was greatest with the combination (*p* < 0.001). Globulin increased and albumin/globulin ratio decreased in treated groups. SOD and GR activities and TAC increased, and MDA decreased in supplemented groups, with the largest responses for the combination, whereas GPx was not significantly affected. In conclusion, Nano-ZnO and SEO, alone or combined, were associated with improved growth performance, nutrient utilization, and redox balance under heat stress. Because treatments were compared by one-way ANOVA, no Nano-ZnO × SEO interaction was tested and the combination is described as a combined effect, not synergy; the higher relative organ weights and lower serum calcium require further evaluation.

## Simple Summary

High temperatures can harm rabbits by slowing growth, reducing feed efficiency, and increasing harmful stress in the body. This study tested whether adding nano zinc oxide and a natural sesame oil extract to rabbit diets could reduce these negative effects. Eighty young rabbits were kept under hot conditions and fed either a normal diet or diets with these supplements, alone or combined. Rabbits receiving the supplements grew faster, used feed more efficiently, and showed better digestion than those on the standard diet. They also had stronger natural defenses that protect their bodies from damage. The combined treatment gave the best overall results. These findings suggest that using safe dietary supplements can help improve rabbit health and productivity during hot weather, supporting farmers facing rising temperatures.

## 1. Introduction

Heat stress (HS) is one of the most critical environmental stressors affecting livestock production systems, particularly in hot regions such as Upper Egypt, where it severely compromises animal welfare, physiological stability, health status, and growth performance during the summer season [1]. The growing importance of rabbit production in the global meat industry has heightened concerns regarding thermal stress, as elevated ambient temperatures adversely impact productivity, meat quality, and survival rates in rabbits [2,3,4]. The optimal temperature for efficient rabbit production ranges between 15 and 25 °C, while the recommended relative humidity for post-weaning rabbits is 55–65%. However, when ambient temperature exceeds 30 °C, rabbits experience heat stress, and at temperatures above 35 °C, their thermoregulatory capacity becomes insufficient, potentially leading to fatal heat exhaustion [5,6,7]. The detrimental effects of HS are associated with profound physiological and biochemical alterations, including impaired immune responses, increased generation of reactive oxygen species (ROS), and enhanced lipid peroxidation in cellular membranes [8,9,10]. Additionally, elevated environmental temperatures suppress the hypothalamic secretion of thyroid-stimulating hormones, resulting in reduced circulating thyroid hormones, which consequently lowers metabolic rate and endogenous heat production in rabbits [11]. These physiological disruptions increase the nutritional demands of animals, particularly for essential nutrients and antioxidants, to mitigate oxidative damage and maintain productive performance under stress conditions [12]. To alleviate HS-induced adverse effects, various nutritional strategies have been explored, including supplementation with nanoparticles of minerals and vitamins [13,14], prebiotics and probiotics [15,16], phytogenic compounds [17], and other functional feed additives. Among essential trace minerals, zinc plays a pivotal role in growth, protein synthesis, antioxidant defense, gut integrity, and metabolic regulation [18,19]. Recently, nanotechnology has enabled the development of nanoparticle-based mineral supplements with unique physicochemical properties, such as increased surface area and bioavailability, which may enhance their biological efficacy compared to conventional sources [20,21]. Zinc oxide nanoparticles (Nano-ZnO), typically ranging from 1 to 100 nm in size, have been reported to improve growth performance, nutrient digestibility, immune function, and gut microbiota across livestock species, including rabbits, when included in diets at levels between 20 and 100 mg/kg [22,23,24,25,26,27]. Dietary phytate, primarily contributed by cereal and oilseed ingredients, may reduce zinc absorption through the formation of poorly soluble complexes [28]. Sesame (*Sesamum indicum* L.), a member of the Pedaliaceae family, contains several bioactive constituents. Whole sesame seeds contain oil, protein, minerals, vitamins, phytate, and lignans, whereas sesame-derived lipid fractions have been reported to contain lipophilic compounds such as sesamin, sesamolin, sesamol, tocopherols, and unsaturated fatty acids [29,30,31]. These constituents have been associated with antioxidant and physiological activities. However, the composition of sesame-derived oils may vary according to the raw material and extraction procedure, and the specific commercial SEO batch used in the present study was not independently characterized.

Although Nano-ZnO and sesame-derived oils have been evaluated separately as feed additives, information on their combined use in growing rabbits exposed to heat stress remains limited. Therefore, the present study evaluated Nano-ZnO and sesame essential oil (SEO) individually and in combination under naturally occurring summer conditions. It was therefore hypothesized that dietary supplementation with Nano-ZnO and SEO, administered individually or in combination, could alleviate heat stress-induced oxidative imbalance and improve productive performance in growing rabbits. Accordingly, the present study aimed to evaluate the effects of Nano-ZnO, SEO, and their combined application on growth performance, carcass traits, nutrient digestibility, mineral retention, and antioxidant status in growing rabbits exposed to heat stress.

## 2. Materials and Methods

### 2.1. Preparation and Characterization of Zinc Oxide Nanoparticles

**Figure 1:**
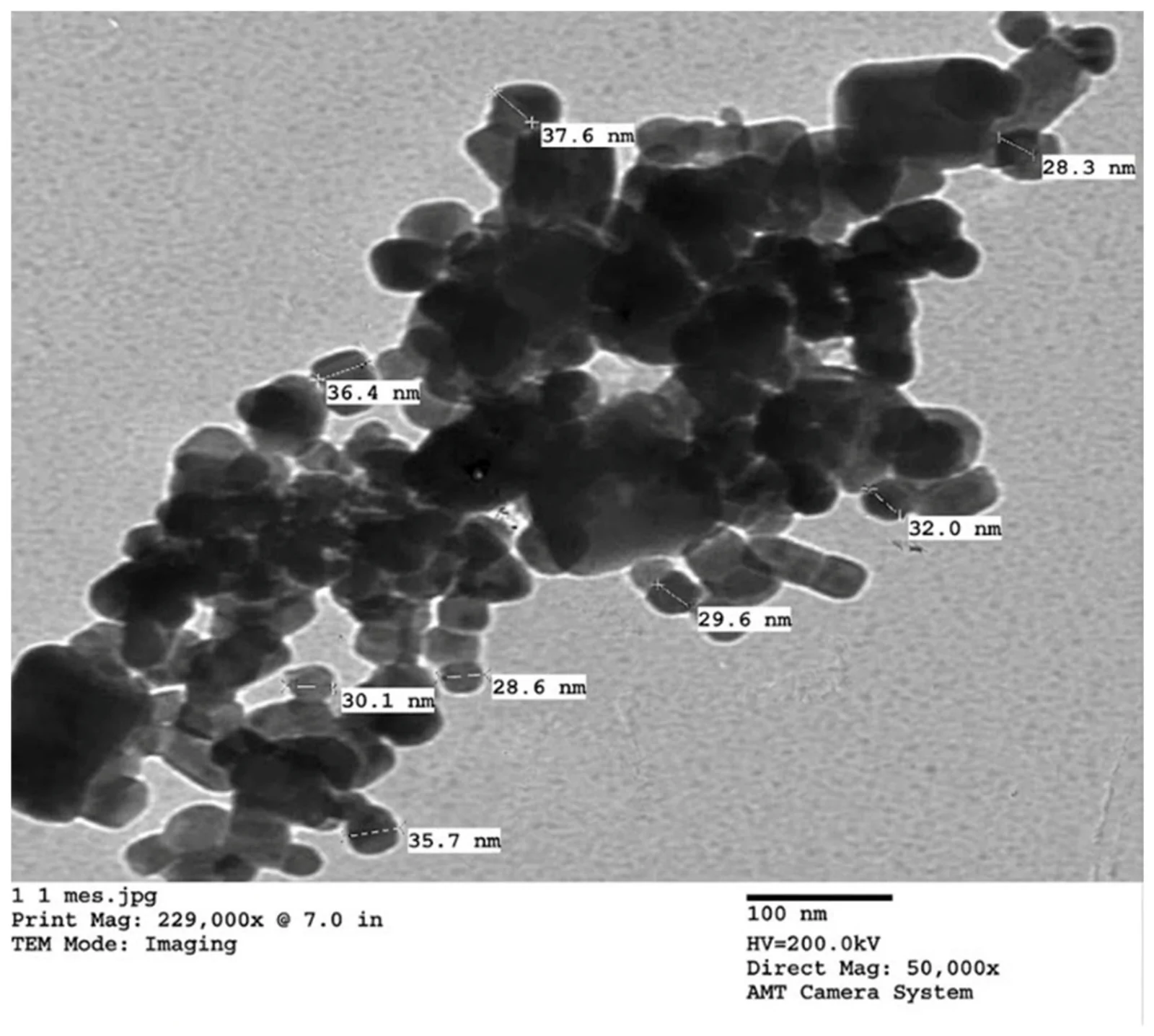
Transmission electron microscopy (TEM) micrograph of biosynthesized zinc oxide nanoparticles (Nano-ZnO), displaying predominantly spherical morphology and a fairly uniform distribution. The nanoparticle size ranges around an average diameter of approximately 32 nm, based on the observed measurements.

Zinc oxide nanoparticles (Nano-ZnO) were biosynthesized using a zinc carbonate precursor in combination with culture filtrates of *Aspergillus niger*. Briefly, fungal biomass was cultured in potato dextrose broth, washed thoroughly, and subsequently incubated in distilled water to obtain a cell-free filtrate. This filtrate was then mixed with the zinc carbonate precursor suspension under controlled alkaline conditions (pH 10–12) and temperature to initiate the precipitation of zinc hydroxide. The resulting precipitate was digested, centrifuged, dried, and calcined at approximately 450 °C to yield Nano-ZnO, following the procedure described by Tamim et al. [32]. The synthesized nanoparticles were characterized by transmission electron microscopy (TEM) (JEM-2100 F, JEOL Ltd., Tokyo, Japan) to determine particle size and morphology; surface structure was not evaluated, and no surface-specific characterization such as BET surface area analysis or X-ray diffraction was performed. Image analysis of representative fields (*N* = 100) revealed a mean particle diameter of approximately 32.3 ± 3.6 nm with a size distribution ranging from 28.3 nm to 37.6 nm (Figure 1).

### 2.2. Experimental Animals and Design

The experiment was conducted at the Rabbit Research Unit, Department of Animal and Poultry Production, Qena University, Egypt. A total of 80 male V-line growing rabbits (45 days old; average body weight 1100 ± 55 g) were randomly assigned to four experimental groups (*n* = 20 per group), with each group consisting of 10 replicates and two rabbits per replicate. The rabbits were obtained from Al-Hussein Rabbit Farm (Suez, Egypt). Only male rabbits were used in order to remove sex as a source of variation in growth rate, feed intake, nutrient utilization, and serum biochemical concentrations, and the growing phase (45–105 days of age) was selected because it is the commercially decisive fattening stage, during which nutrient requirements are high and thermoregulatory capacity is still developing, so that animals at this stage are particularly susceptible to elevated ambient temperature and humidity. Sesame essential oil (SEO) was purchased from EL Masrayia Company for the Extraction of Natural Oils, Cairo, Egypt. No independent compositional analysis of the SEO batch used in the present study was conducted. However, published literature indicates that sesame-derived oils contain bioactive constituents, including lignans such as sesamin, sesamolin, and sesamol, together with tocopherols and unsaturated fatty acids, which have been associated with antioxidant and physiological activities [29,30,31]. Therefore, the biological effects observed in the present study are discussed with reference to the reported properties of sesame-derived oils rather than to the measured composition of the specific SEO batch used.

The experimental design included a control group fed a basal diet without supplementation, a Nano-ZnO group receiving the basal diet supplemented with nano zinc oxide at 60 mg/kg diet, a sesame essential oil (SEO) group receiving the basal diet supplemented with sesame essential oil at 300 mg/kg diet, and a combination group receiving the basal diet supplemented with both nano zinc oxide (60 mg/kg diet) and SEO (300 mg/kg diet). The supplements (Nano-ZnO, SEO and their combination) were added on top of the formulated basal diet without displacing any primary dietary components. The supplementation levels were selected from the ranges previously reported to be biologically active in rabbits and other livestock species, namely 20–100 mg Nano-ZnO/kg diet for zinc oxide nanoparticles [22,23,24,25,26,27] and practical inclusion levels for sesame-derived oils in rabbit diets. Nano-ZnO was therefore not evaluated as a correction of a dietary zinc deficiency: the basal diet was formulated to meet the zinc requirement of growing rabbits [33], and the supplemental nano-sized zinc was tested as a functional additive whose physiological activity may differ from that of conventional inorganic zinc sources.

The supplements were weighed, pre-mixed with a portion of the basal mash to form a premix, and then thoroughly homogenized into the complete dry basal mash before pelleting, so that the additives were incorporated into the feed matrix rather than surface-applied to finished pellets. All four experimental diets were manufactured using the same pellet mill and under identical feed-processing conditions and were pelleted before feeding. However, the conditioning and pelletizing temperatures were not recorded during feed manufacturing and therefore cannot be reported. Consequently, the potential effects of pelleting temperature on the stability of SEO bioactive compounds could not be evaluated and should be considered a limitation of the present study.

Rabbits were provided *ad libitum* access to feed and water throughout the 60-day experimental period. The composition and chemical analysis of the basal diet are shown in Table 1, and the formulation was designed to meet the nutritional requirements of growing rabbits [33]. Animals were housed in galvanized wire mesh cages (50 × 35 × 45 cm) equipped with manual feeders and automatic nipple drinkers. The housing facility was managed under standard experimental husbandry conditions with a photoperiod of 16 h light and 8 h dark; ambient temperature and relative humidity inside the building were not artificially regulated and followed the prevailing natural summer conditions. Ambient temperature and relative humidity were monitored daily at cage level throughout the experimental period.

### 2.3. Environmental Conditions

The study was conducted during the summer season of 2025. Ambient temperature (dry bulb temperature, °C) and relative humidity (%) were monitored using a digital thermo-hygrometer (Model HTC-2, Guangzhou Rongtest Electronics Co., Ltd., Guangzhou, China). The instrument was positioned at cage level within the rabbit housing facility to reflect the environmental conditions experienced by the animals. Temperature and relative humidity measurements were recorded daily, and 10-day period averages were calculated. The temperature–humidity index (THI) was determined according to the equation of Marai et al. [34], which was adopted in preference to the THI formulations developed for cattle and poultry because it was derived specifically for rabbits and is accompanied by a rabbit-specific classification of thermal load, allowing the recorded values to be interpreted directly against published stress categories for this species:

THI = Tdb − [(0.31 − 0.31 × RH) × (Tdb − 14.4)]

Based on THI values, heat stress was categorized as follows: no stress (THI < 27.8), moderate (27.8–28.8), severe (28.9–29.9), and very severe (>30.0) [34]. According to the calculated THI (Table 2), the maximum daily THI averaged over the six 10-day periods ranged from 29.47 to 30.58, placing the daily thermal peaks in the severe to very severe categories, whereas the corresponding minimum daily THI ranged from 22.27 to 23.99, which falls below the no-stress threshold. Rabbits were therefore exposed to a daily cycle in which the thermal load reached severe to very severe levels, rather than to a constant heat load. Because THI integrates relative humidity as well as dry-bulb temperature, this degree of thermal stress was reached at maximum ambient temperatures below 35 °C, and heat stress in the present study is accordingly classified on the basis of THI rather than on a single ambient-temperature threshold.

### 2.4. Growth Performance

Body weight (BW) was recorded on an individual basis at 45 (initial), 65, 85, and 105 days of age. Feed intake (FI) was determined per replicate cage as the difference between the quantity of feed offered and the residual feed recovered, with feed offered and refusals weighed throughout each interval, and was then summed and expressed per rabbit for each of the 45–65, 65–85, and 85–105 day intervals and for the overall 45–105 day period; FI was therefore not derived from single-day measurements taken only on weighing days. Body weight gain (BWG) and feed conversion ratio (FCR) were calculated accordingly as indicators of growth performance.

### 2.5. Digestibility Trial

A digestibility trial was conducted during the last five days of the experiment. Ten rabbits from each treatment (one per replicate) were randomly selected and housed individually in metabolic cages designed for separate collection of feces and urine. The trial was positioned in the final five days of the feeding period so that the excreta collected reflected nutrient utilization after sustained exposure to the experimental diets and were not influenced by the pre-experimental diet; consequently, the digestibility data characterize the late supplementation phase, and any differences in nutrient utilization occurring during the early phase of supplementation would not have been captured. A feed-withdrawal period of 24 h was applied before the start and after the end of the collection period in order to standardize gastrointestinal fill, to complete evacuation of feed consumed before the collection window, and to mark the end of transit for feed consumed during it, thereby limiting carry-over error in the total-collection calculation. Water was available ad libitum at all times during feed withdrawal. It is nevertheless acknowledged that feed withdrawal of this duration constitutes a metabolic challenge for growing rabbits and may itself influence measured nutrient digestibility, and this is recognized as a methodological limitation of the present protocol. Temperature and relative humidity during the digestibility trial were recorded daily at cage level using the digital thermo-hygrometer described in Section 2.3.

During the 5-days collection period, the average daily minimum and maximum temperature were 24.80 °C and 33.32 °C, respectively, with the relative humidity range of 41.35–56.73%. The THI ranged from 23.41 (minimum THI) to 29.88 (maximum THI).

Daily feed intake was recorded, and total fecal output was collected, weighed, and stored at −20 °C until analysis. Contaminants such as feed residues and hair were carefully removed. Fecal samples were dried at 60 °C for 48 h and analyzed for moisture, ash, crude protein (CP), and ether extract (EE) using standard AOAC methods (930.15, 942.05, 984.13, and 954.02, respectively) [35].

Apparent nutrient digestibility (%) was calculated as:

Apparent digestibility (%)=[(Nutrient intake−Nutrient in feces)÷Nutrient intake]×100

### 2.6. Carcass Traits

At the end of the experiment (105 days), ten rabbits per group (one per replicate) were randomly selected, fasted overnight with free access to water, and slaughtered according to approved ethical standards. Dressing percentage was calculated after removal of the head, digestive tract, and internal organs. The weights of liver, lungs, heart, kidneys, cecum, small intestine, and head were recorded and expressed relative to live body weight.

### 2.7. Blood Sampling and Biochemical Analysis

At the end of the experimental period, blood samples (approx. 3 mL) were collected from the marginal ear vein immediately prior to slaughter, into plain tubes without anticoagulant of ten rabbits per treatment group (one per replicate). Samples were centrifuged at 3000 rpm for 10 min to obtain serum, which was stored at −20 °C until analysis. Serum concentrations of total protein, albumin, cholesterol, urea, and creatinine were determined using commercial diagnostic kits (Spectrum Chemical Company, Cairo, Egypt). Globulin concentration was calculated by subtracting albumin from total protein. Malondialdehyde (MDA), total antioxidant capacity (TAC), superoxide dismutase (SOD), glutathione peroxidase (GPx), and glutathione reductase (GR) were determined spectrophotometrically using commercial colorimetric assay kits (Biodiagnostic Co., Dokki, Giza, Egypt) according to the manufacturer’s instructions.

### 2.8. Mineral Analysis

Liver samples from ten rabbits per treatment were collected at slaughter and stored at −20 °C for mineral analysis. Serum samples were also analyzed for mineral concentrations. Zinc (Zn), magnesium (Mg), and calcium (Ca) levels in feed, serum, and liver were determined using atomic absorption spectrophotometry (PerkinElmer Analyst 800, Shelton, CT, USA). Phosphorus (P) concentration was measured colorimetrically following AOAC guidelines [35].

### 2.9. Statistical Analysis

Data were analyzed using a completely randomized design (CRD) with the General Linear Model (GLM) procedure of SAS software (version 9.2) [36], considering replicate as the experimental unit. One-way analysis of variance (ANOVA) was conducted, and differences among means were compared using Duncan’s multiple range test. The model contained the single fixed effect of dietary treatment; the design was not factorial, and no Nano-ZnO × SEO interaction term was therefore fitted or tested. Statistical significance was declared at *p* < 0.05, while values between 0.05 and 0.10 were considered as trends. Data normality was assessed using the Anderson–Darling test. Graphs were generated using GraphPad Prism software (version 9).

## 3. Results

### 3.1. Environmental Conditions

The environmental conditions recorded during the experimental period are presented in Table 2. The ambient temperature ranged from 23.42 to 34.12 °C, with relative humidity between 38.85% and 59.71%. The 10-day period averages of the maximum daily temperature–humidity index (THI) ranged from 29.47 to 30.58, corresponding to the severe and very severe heat-stress categories, whereas the 10-day period averages of the minimum daily THI ranged from 22.27 to 23.99. Rabbits were therefore exposed throughout the study to a daily thermal cycle whose peak reached severe to very severe heat-stress levels.

### 3.2. Growth Performance

No mortality was observed throughout the experimental period. The effects of dietary supplementation with Nano-ZnO, SEO, and their combination on the growth performance of growing rabbits are summarized in Table 3. Body weight (BW) was significantly influenced by dietary treatments. Rabbits fed diets supplemented with Nano-ZnO or SEO alone exhibited significantly higher BW than the control group at 65 days of age (*p* = 0.001), whereas the combination group did not differ significantly from the control. At 85 and 105 days of age, all supplemented groups (Nano-ZnO, SEO, or their combination) showed significantly higher BW compared with the control group (*p* = 0.001). Body weight gain (BWG) responded differently across the individual intervals. During the 45–65 day interval, BWG was significantly higher than in the control group in the Nano-ZnO group (*p* = 0.001), whereas the SEO and combination groups did not differ significantly from the control. During the 85–105 day interval, BWG was significantly higher than in the control group in the Nano-ZnO and combination groups (*p* = 0.007), whereas the SEO group did not differ significantly from the control. Over the overall 45–105 day period, BWG was significantly higher than in the control group in all three supplemented groups (*p* = 0.001), the highest value being recorded for Nano-ZnO. During the 65–85 day interval, BWG did not differ significantly among groups (*p* = 0.083); the higher values observed in the treated groups, particularly in the Nano-ZnO group, therefore represent numerical differences only. Feed intake (FI) was also affected by dietary supplementation. Rabbits in the Nano-ZnO group showed significantly higher FI compared with the control at all measured intervals (*p* < 0.05). In contrast, the SEO group recorded the lowest FI during the early growth phase (45–65 days) (*p* < 0.01), indicating a possible effect on feed consumption patterns. Feed conversion ratio (FCR) was significantly improved in rabbits receiving Nano-ZnO, SEO, and their combination compared to the control group during the 45–65-day period (*p* < 0.01) and across the overall experimental period (45–105 days) (*p* = 0.009). No significant differences in FCR were observed during the intermediate growth phases (65–85 and 85–105 days). Overall, dietary supplementation enhanced feed efficiency, with treated groups demonstrating lower FCR values than the control.

### 3.3. Nutrient Digestibility

The effects of dietary supplementation with Nano-ZnO, SEO, and their combination on nutrient digestibility are presented in Table 4. Dry matter (DM) and crude protein (CP) digestibility were significantly influenced by dietary treatments. Rabbits receiving SEO either alone or in combination with Nano-ZnO exhibited significantly higher DM and CP digestibility compared with the control group (*p* < 0.01). In addition, Nano-ZnO supplementation alone also improved CP digestibility relative to the control (*p* < 0.01). Ether extract (EE) digestibility was markedly enhanced by the combined supplementation of Nano-ZnO and SEO, showing the highest value among all groups (*p* < 0.01), indicating a potential combined effect between the two additives. In contrast, EE digestibility in the Nano-ZnO and SEO groups did not differ significantly from the control. No significant differences were observed in crude fiber (CF) digestibility among the experimental groups (*p* = 0.458), suggesting that dietary treatments had minimal impact on fiber utilization.

### 3.4. Carcass Characteristics

The effects of dietary Nano-ZnO, SEO, and their combination on carcass traits are summarized in Table 5. No significant differences were detected among treatment groups in dressing percentage or the relative weights of lungs, heart, small intestine, and cecum (*p* > 0.05). However, relative liver and kidney weights were significantly affected by dietary treatments. Rabbits fed the combined Nano-ZnO and SEO diet exhibited significantly higher relative liver weight compared with the Nano-ZnO, SEO, and control groups (*p* = 0.001). Similarly, relative kidney weight was significantly increased in the combination group compared with the control and Nano-ZnO groups (*p* = 0.018), while the SEO group showed intermediate values. The remaining carcass components were not significantly influenced by dietary supplementation, although a tendency toward increased head weight was observed in the SEO and combination groups (*p* = 0.057).

### 3.5. Serum Mineral Concentrations

The effects of dietary supplementation with Nano-ZnO, SEO, and their combination on serum mineral concentrations are presented in Table 6. Serum zinc (Zn) concentration was significantly influenced by dietary treatments (*p* < 0.001). Rabbits supplemented with Nano-ZnO exhibited the highest Zn levels compared with all other groups, followed by the combination group, while the lowest Zn concentration was observed in the SEO group. Similarly, serum magnesium (Mg) levels were significantly affected (*p* < 0.001), with the Nano-ZnO group showing the highest values, followed by the control and SEO groups. In contrast, the combination treatment resulted in the lowest Mg concentration.

Phosphorus (P) concentration differed significantly among treatments (*p* < 0.001), with the highest level detected in rabbits fed the combined Nano-ZnO and SEO diet. Both the control and SEO groups showed intermediate values, whereas the Nano-ZnO group exhibited the lowest P concentration. Serum calcium (Ca) concentration was also significantly altered by dietary supplementation (*p* < 0.001). The control group recorded the highest Ca levels, followed by the SEO group, while both Nano-ZnO and combination treatments resulted in significantly reduced Ca concentrations, with the lowest value observed in the combination group.

### 3.6. Serum Biochemical Parameters

The effects of dietary supplementation with Nano-ZnO, SEO, and their combination on serum biochemical parameters are presented in Table 7. Serum total protein and albumin concentrations were not significantly affected by dietary treatments (*p* > 0.05), although numerically higher values of total protein were observed in the supplemented groups compared with the control. In contrast, globulin concentration was significantly influenced by dietary supplementation (*p* = 0.004). Rabbits receiving Nano-ZnO, SEO, or their combination exhibited higher globulin levels than the control group, with no significant differences among the supplemented treatments. Consequently, the albumin-to-globulin (A/G) ratio was significantly reduced in all treated groups compared with the control (*p* = 0.001). Total cholesterol levels were not significantly altered by dietary treatments (*p* = 0.139), although a numerical increase was observed in the SEO and combination groups. Serum creatinine concentration was significantly affected by supplementation (*p* = 0.001). The SEO group showed significantly lower creatinine levels compared with the control, Nano-ZnO, and combination groups, which did not differ significantly from each other. No significant differences were observed in serum urea levels among the experimental groups (*p* = 0.635).

### 3.7. Antioxidant Status

**Figure 2:**
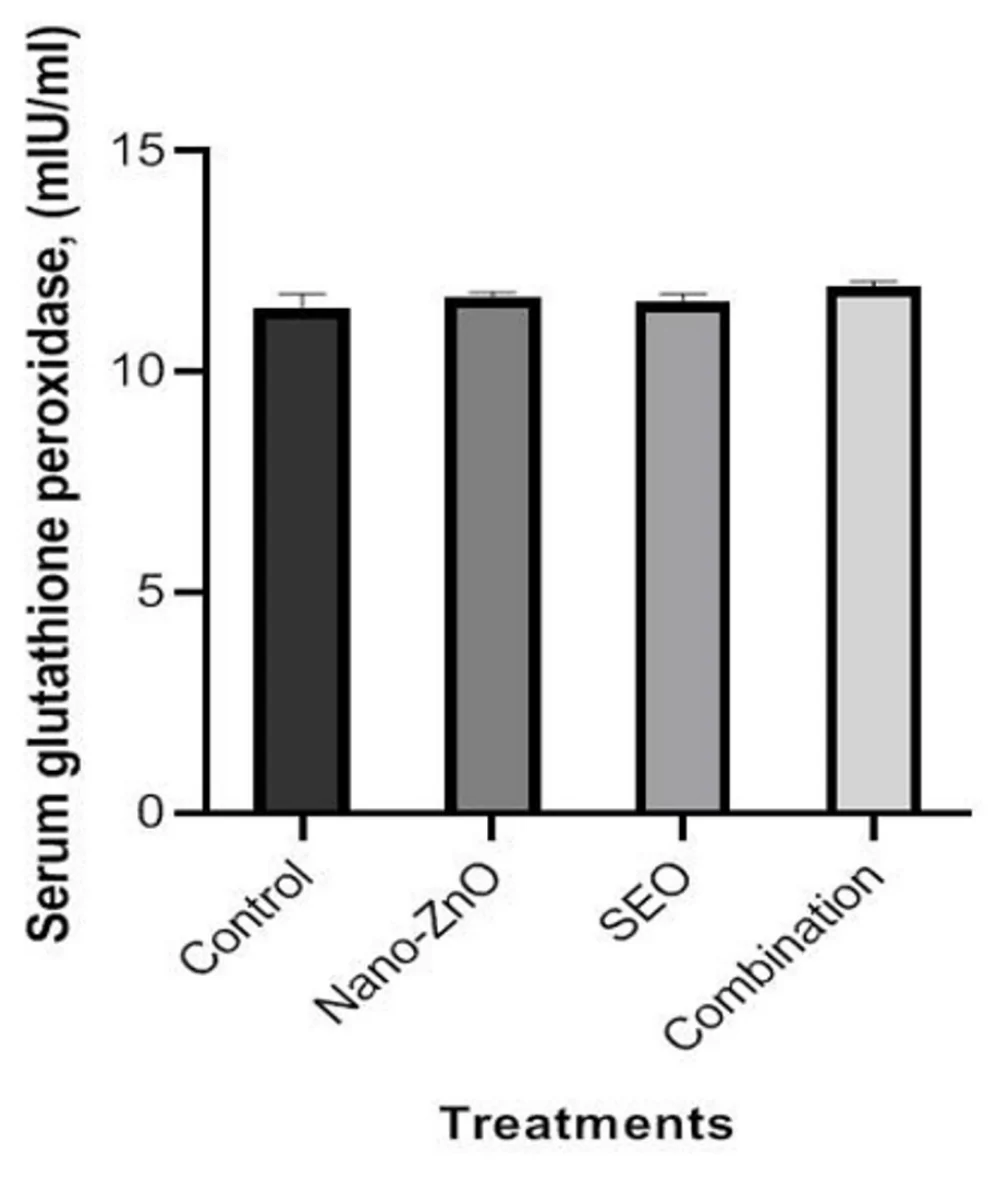
Effects of dietary nano-zinc oxide (Nano-ZnO), sesame essential oil (SEO), and their combination on serum glutathione peroxidase (GPx) activity in growing rabbits exposed to heat stress. Values are expressed as mean ± SEM (*n* = 10). GPx activity did not differ significantly among treatments (*p* > 0.05); differences between groups therefore represent numerical variation only.

**Figure 3:**
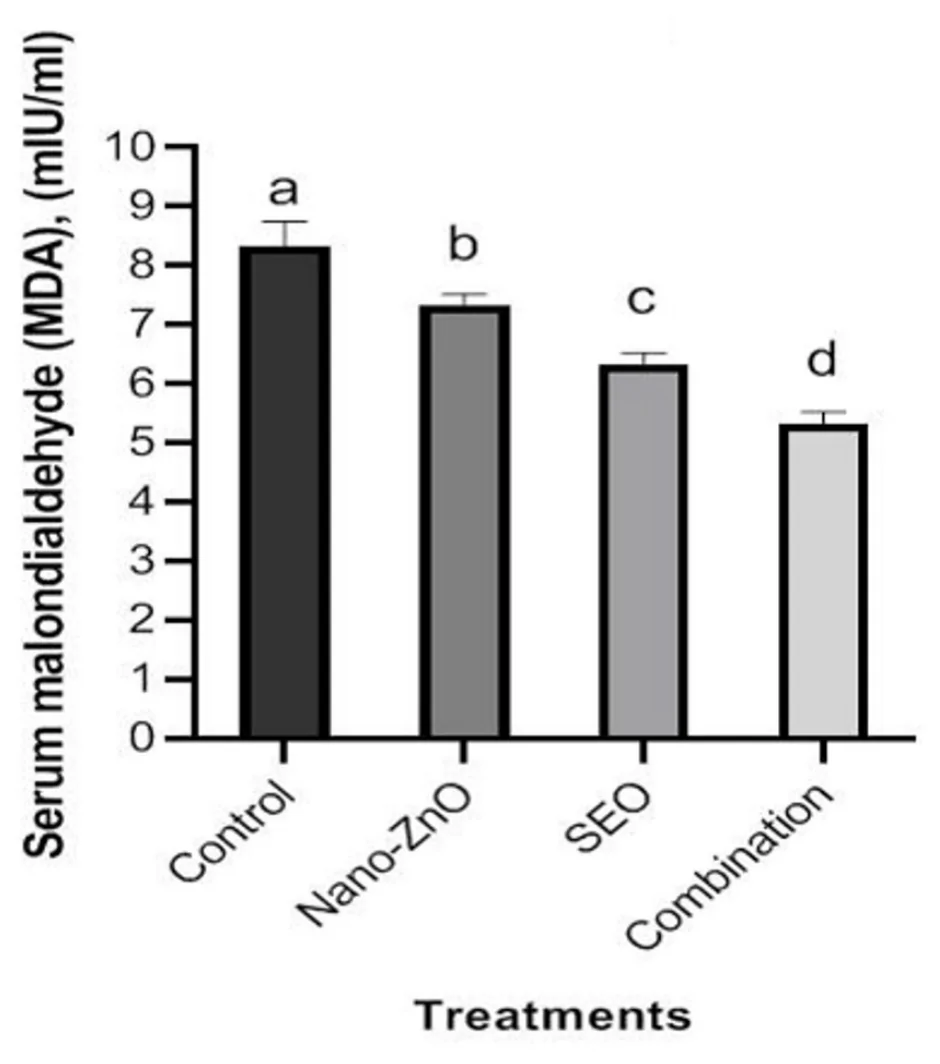
Effects of dietary nano-zinc oxide (Nano-ZnO), sesame essential oil (SEO), and their combination on serum malondialdehyde (MDA) concentration in growing rabbits exposed to heat stress. Values are expressed as mean ± SEM (*n* = 10). Different superscripts indicate significant differences (*p* < 0.05).

**Figure 4:**
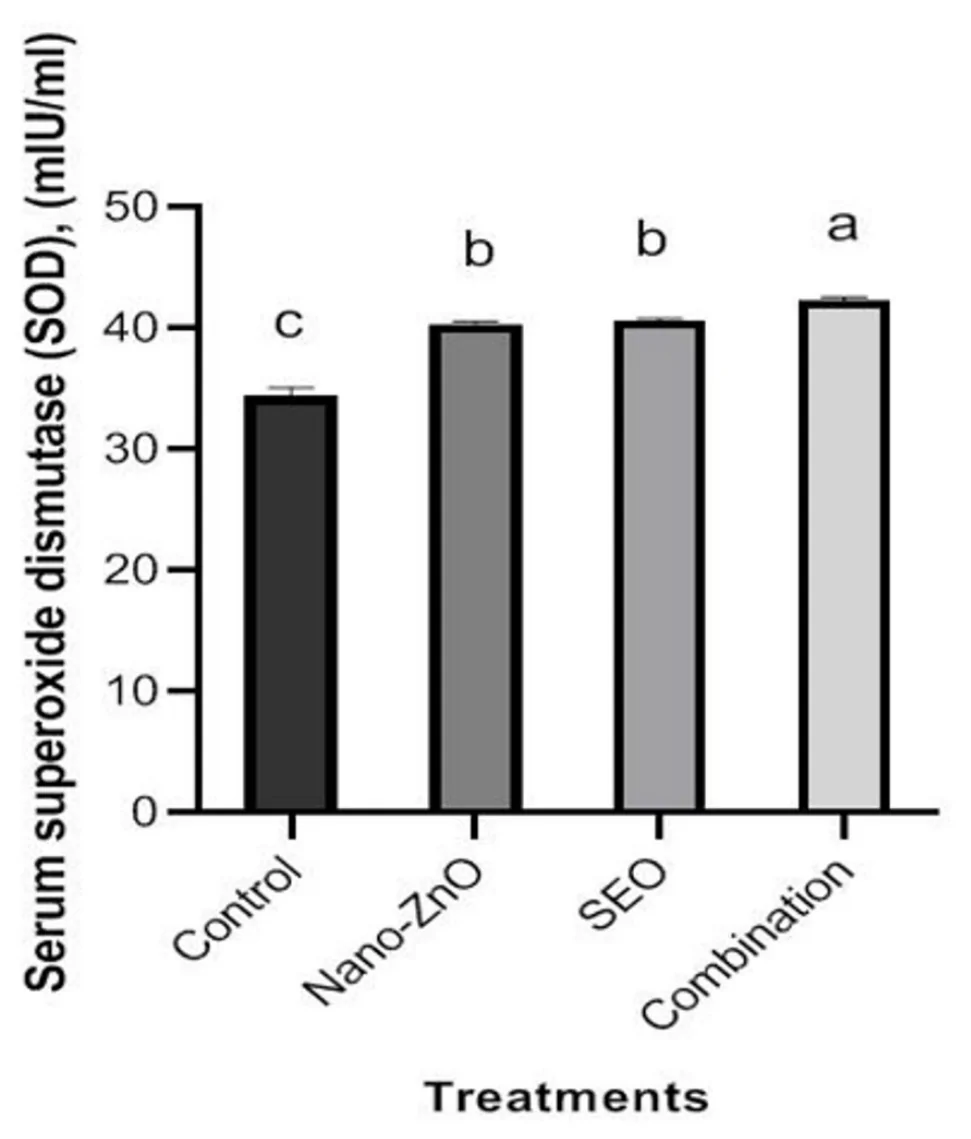
Effects of dietary nano-zinc oxide (Nano-ZnO), sesame essential oil (SEO), and their combination on serum superoxide dismutase (SOD) activity in growing rabbits exposed to heat stress. Values are expressed as mean ± SEM (*n* = 10). Different superscripts indicate significant differences (*p* < 0.05).

**Figure 5:**
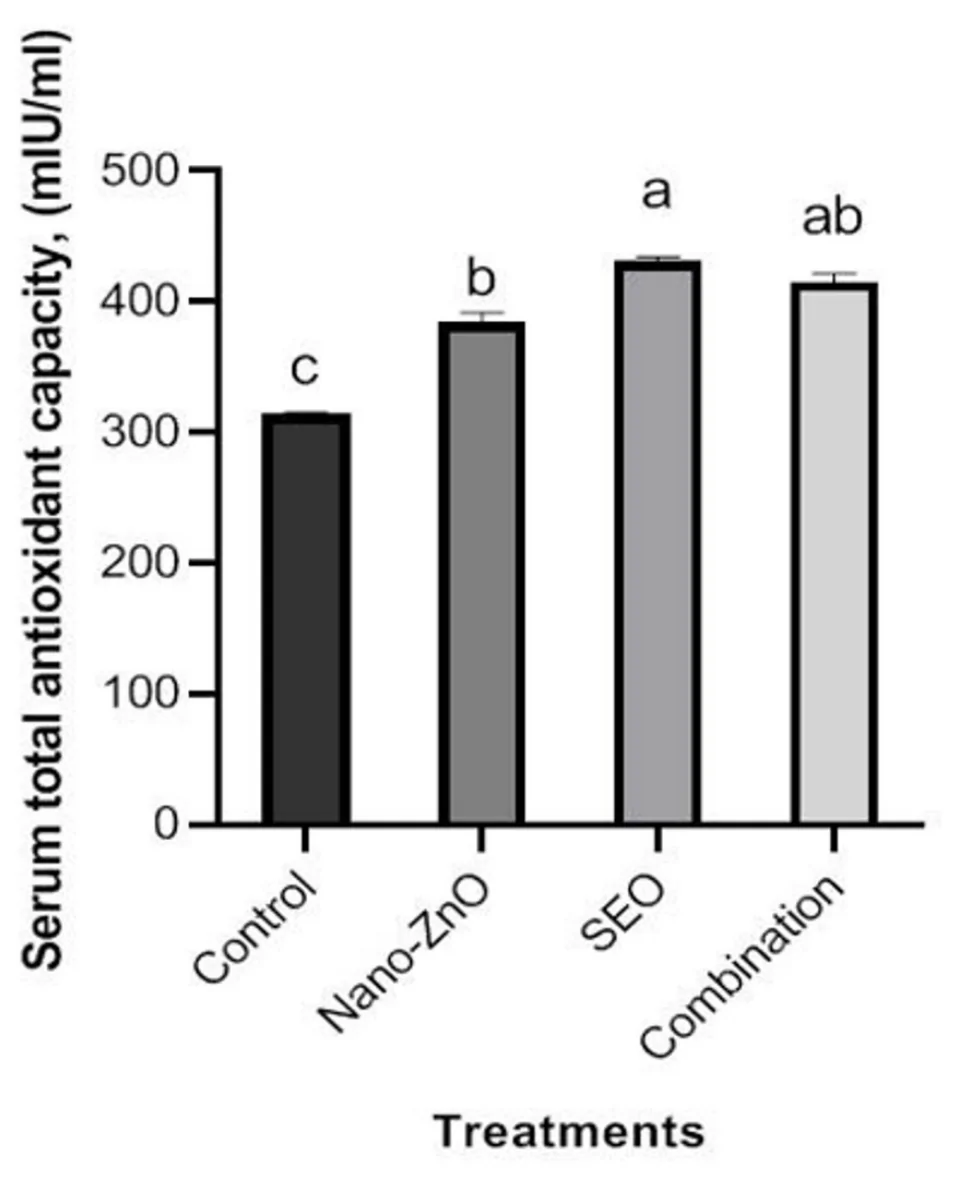
Effects of dietary nano-zinc oxide (Nano-ZnO), sesame essential oil (SEO), and their combination on serum total antioxidant capacity (TAC) in growing rabbits exposed to heat stress. Values are expressed as mean ± SEM (*n* = 10). Different superscripts indicate significant differences (*p* < 0.05).

**Figure 6:**
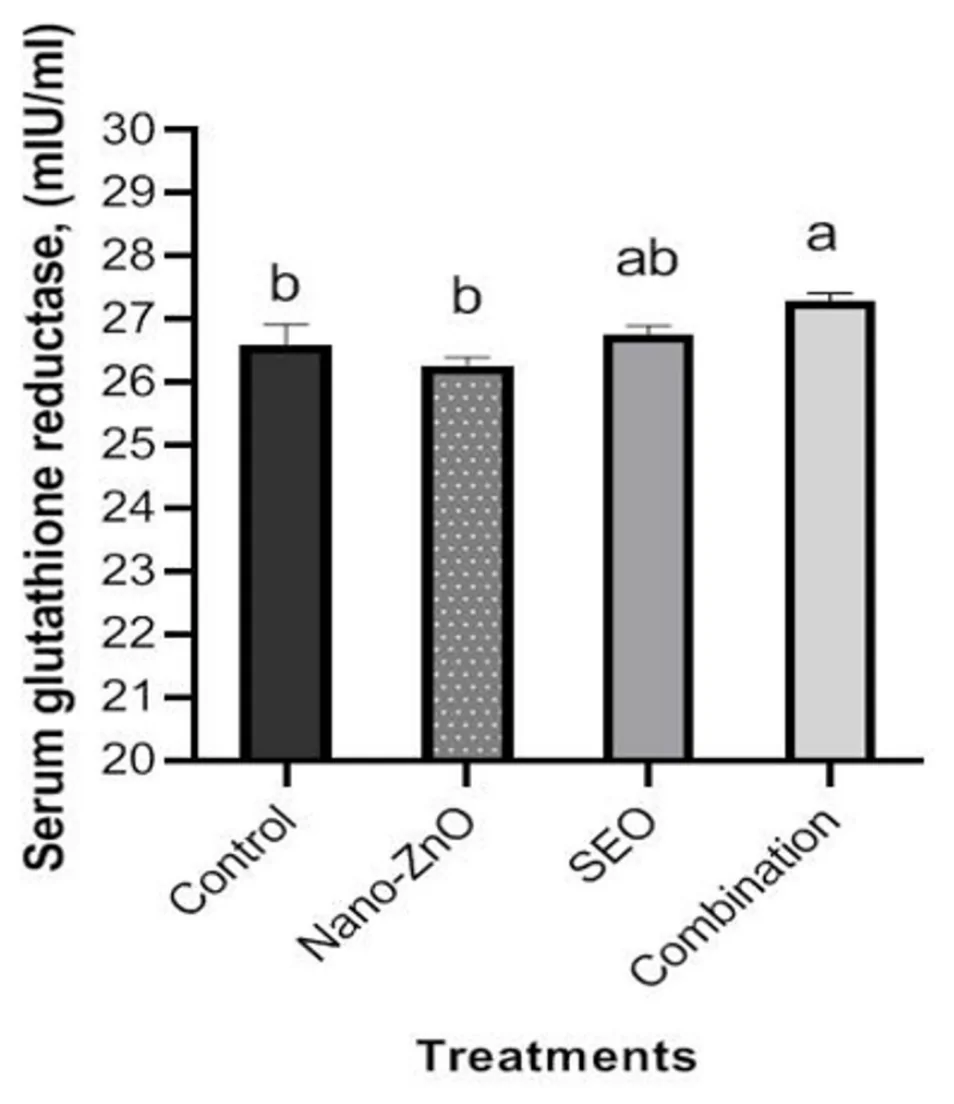
Effects of dietary nano-zinc oxide (Nano-ZnO), sesame essential oil (SEO), and their combination on serum glutathione reductase (GR) activity in growing rabbits exposed to heat stress. Values are expressed as mean ± SEM (*n* = 10). Different superscripts indicate significant differences (*p* < 0.05).

The effects of dietary Nano-ZnO, SEO, and their combination on serum antioxidant indicators are presented in Figure 2, Figure 3, Figure 4, Figure 5 and Figure 6. Glutathione peroxidase (GPx) activity was non-significantly influenced by dietary treatments (Figure 2). Malondialdehyde (MDA) levels, an indicator of lipid peroxidation, were significantly reduced in all supplemented groups compared with the control (Figure 3). The lowest MDA concentration was recorded in rabbits receiving the combined treatment, indicating a marked reduction in oxidative stress. Superoxide dismutase (SOD) activity was significantly enhanced by dietary supplementation (Figure 4). Both Nano-ZnO and SEO treatments increased SOD activity, while their combination resulted in the greatest elevation compared with the control group. Total antioxidant capacity (TAC) showed a similar trend (Figure 5), with significantly higher values observed in all treated groups relative to the control. The combination treatment demonstrated superior antioxidant capacity compared with individual supplementation. In addition, glutathione reductase (GR) activity was significantly increased in response to dietary treatments (Figure 6). The combined Nano-ZnO and SEO group exhibited the highest GR activity, followed by the individual treatments, whereas the control group showed the lowest values. Overall, these findings indicate that Nano-ZnO and SEO supplementation, particularly in combination, effectively enhanced the antioxidant defense system in heat-stressed rabbits.

### 3.8. Liver Zinc Concentration

**Figure 7:**
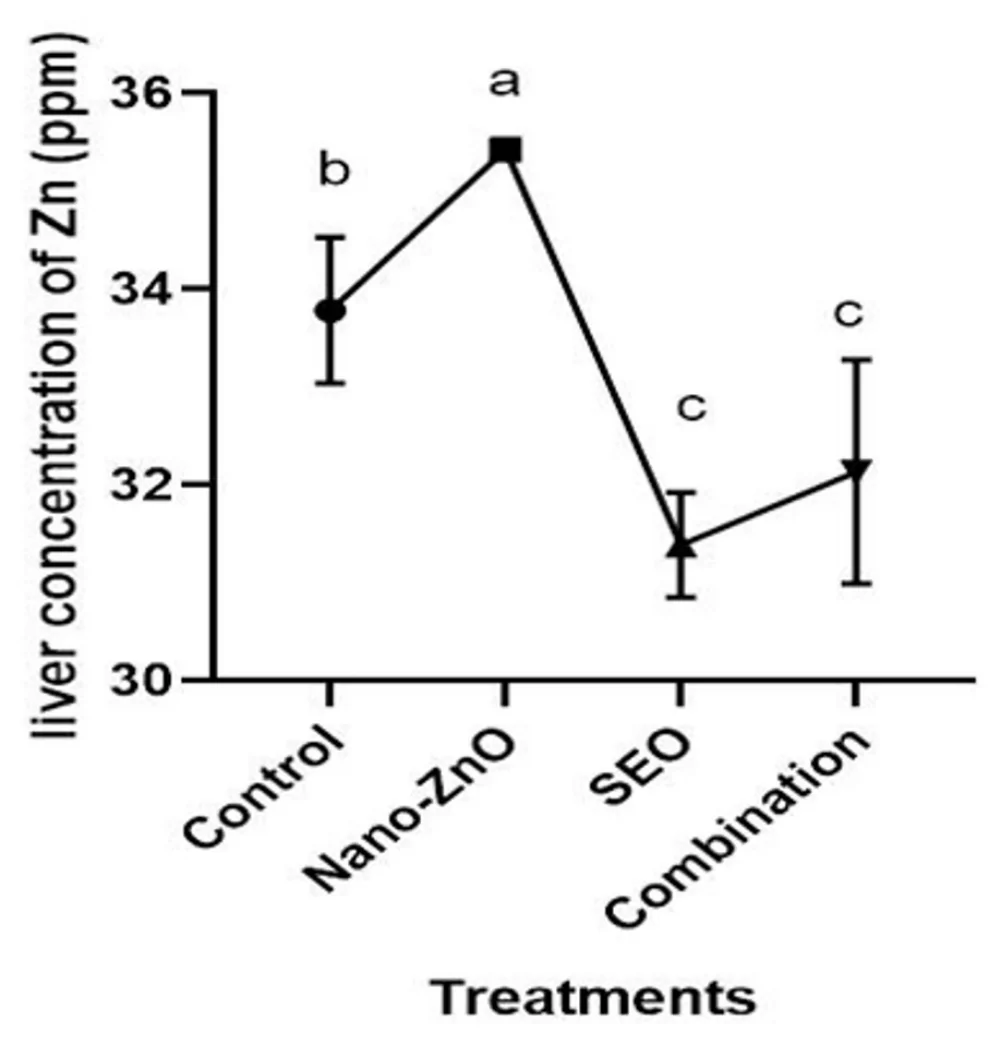
Effects of dietary nano-zinc oxide (Nano-ZnO), SEO, and their combination on liver zinc (Zn) concentration in growing rabbits. Values are expressed as mean ± SEM (*n* = 10). Different superscripts indicate significant differences (*p* < 0.05).

The effects of dietary treatments on liver zinc concentration are shown in Figure 7. Liver Zn content was significantly affected by dietary supplementation. Hepatic Zn concentration was highest in rabbits fed Nano-ZnO, whereas the SEO and combination groups exhibited significantly lower hepatic Zn concentrations than the control group, the lowest value being recorded in the SEO group (*p* = 0.001).

## 4. Discussion

Heat stress (HS) is a major environmental constraint that adversely affects physiological functions, immune competence, and productivity in rabbits. In the present study, HS severity was confirmed using the temperature–humidity index (THI), which integrates ambient temperature and relative humidity. Based on 10-day period averages THI values (29.47–30.58), rabbits were exposed to severe to very severe heat stress conditions throughout the experimental period. It should be recognized that the physiological responses observed during the 60-day experimental period may have reflected not only the direct effects of sustained heat stress but also progressive physiological acclimation to the prevailing thermal conditions. Prolonged exposure to elevated temperature and humidity may induce adaptive changes in feed intake, metabolic activity, and thermoregulatory responses, which could modify the magnitude of subsequent physiological responses and potentially influence the apparent efficacy of dietary supplementation. Under such conditions, nutritional interventions are essential to mitigate oxidative stress and maintain productive performance. Zinc is an essential trace element involved in numerous biological processes, particularly under stress conditions, where it plays a critical role in maintaining growth, antioxidant defense, and immune function [24]. In this study, dietary supplementation with Nano-ZnO, SEO, and their combination significantly improved growth performance, as evidenced by increased body weight (BW), body weight gain (BWG), and improved feed conversion ratio (FCR). These improvements may be attributed to the multifunctional role of zinc in enzyme activation, protein synthesis, DNA replication, and cellular proliferation, as well as its contribution to antioxidant protection and immune regulation [37,38,39]. Previous studies have similarly reported that nano-scale zinc oxide enhances growth performance and feed efficiency in rabbits, particularly under heat stress conditions [40,41]. Moreover, supplementation with Nano-ZnO within the range of 30–80 mg/kg has been shown to improve BWG and FCR in heat-stressed rabbits [24,42,43], while zinc in organic or nano forms demonstrates superior bioavailability compared with conventional inorganic sources [44].

The observed performance improvement in rabbits receiving SEO is consistent with previous findings on phytogenic feed additives under thermal stress. Sesame oil is rich in lignans, tocopherols, and unsaturated fatty acids, which possess antioxidant, anti-inflammatory, and metabolic regulatory properties [29,31]. In contrast, the essential minerals and vitamins reported for sesame are concentrated in the whole seed and in the de-oiled meal rather than in the extracted lipid fraction [30], and the present results should therefore not be attributed to a mineral or vitamin contribution from the supplemented oil. For the same reason, phytic acid, which is abundant in sesame seed and meal and is capable of chelating divalent cations, including zinc, and thereby reducing zinc absorption [28], is associated predominantly with the solid seed fraction and would be expected to make a minor contribution through an oil-based additive supplied at 300 mg/kg diet. Antagonism between the sesame-derived additive and dietary zinc is consequently unlikely to explain the present findings, and this expectation is consistent with the higher serum zinc concentration observed when Nano-ZnO and the sesame-derived oil were fed together than in the control group. It must nevertheless be emphasized that the phytate content of the supplemented product was not determined in this study, and this interpretation is contingent on confirmation of the identity and composition of the sesame-derived product. A further observation requiring explanation is that the SEO group exhibited the lowest feed intake during the 45–65-day interval while showing higher dry matter and crude protein digestibility than the control group. These outcomes are not mutually inconsistent: feed intake and the apparent digestibility coefficient are distinct measures, the former quantifying the amount of feed consumed and the latter the proportion of ingested nutrients absorbed. Reduced voluntary intake is a recognized adaptive response that limits metabolic heat production under thermal load, while a lower passage rate at reduced intake, together with the additional dietary lipid supplied by the oil, may increase the proportion of nutrients digested. These mechanisms were not measured in the present study and are offered as plausible explanations rather than demonstrated causes. The enhanced performance observed in the combination group is consistent with a combined effect of Nano-ZnO and SEO, in which nano-sized minerals and bioactive lipophilic phytochemicals may act on complementary pathways of physiological adaptation under HS conditions; this remains an interpretation rather than a demonstrated mechanism, because the study design did not permit an interaction test.

The improvement in nutrient digestibility observed in the present study further supports the growth-enhancing effects of these dietary interventions. Although SEO supplementation was associated with reduced feed intake during the 45-to-65-day interval, this observation is not necessarily inconsistent with the improvement in nutrient digestibility. Feed intake reflects the quantity of feed consumed, whereas digestibility reflects the proportion of ingested nutrients that are absorbed. Under heat stress conditions, reduced voluntary feed intake is a recognized adaptive response that helps limit metabolic heat production. At the same time, a lower feed intake may increase digest retention time within the gastrointestinal tract, thereby enhancing nutrient digestion and absorption. These mechanisms were not directly evaluated in the present study and are therefore presented as plausible explanations rather than confirmed causes. Supplementation with SEO, either alone or in combination with Nano-ZnO, significantly enhanced dry matter (DM) and crude protein (CP) digestibility, while Nano-ZnO improved CP digestibility independently. The combined treatment resulted in the greatest increase in ether extract (EE) digestibility, indicating a combined effect on lipid utilization. Zinc is a cofactor for more than 300 enzymes involved in nutrient metabolism and digestion [45,46], and it enhances the absorption and utilization of proteins, lipids, and carbohydrates [47]. Moreover, zinc supports pancreatic function and digestive enzyme secretion, which may be particularly beneficial under heat stress conditions where digestive efficiency is compromised [48]. These findings are consistent with previous studies reporting improvements in nutrient digestibility in rabbits supplemented with Nano-ZnO or other zinc sources [20,27,44,45,49]. The enhanced EE digestibility observed in the combined treatment may be attributed to the complementary actions of Nano-ZnO and SEO. While Nano-ZnO improves intestinal absorption and enzymatic activity, sesame essential oil provides bioactive compounds that facilitate lipid digestion and absorption. Similar combined effects between nanominerals and phytogenic additives have been reported to improve digestive efficiency and overall performance in heat-stressed animals [20,50].

Carcass trait analysis revealed that most parameters were not significantly affected by dietary treatments, indicating that supplementation did not alter carcass composition. However, relative liver and kidney weights were higher in the combination group. Increased relative weights of these organs may reflect a treatment-related physiological response involving altered nutrient and mineral handling, since the liver is central to nutrient metabolism, lipid processing, antioxidant defense, and biotransformation, and the kidney to mineral homeostasis and excretion. Their biological significance cannot be determined from organ weight alone, because increased organ mass may also arise from pathological processes such as inflammation, congestion, or hypertrophy. No histopathological examination and no organ-specific functional assay were performed in the present study, and serum urea and creatinine remained within the range recorded for the control group, which is not by itself sufficient evidence of preserved hepatic or renal integrity. The increased relative organ weights are therefore reported as a treatment-related observation of undetermined biological significance, and no conclusion regarding organ health or safety is drawn from the present data. Enlarged visceral organs may also raise basal metabolic demand, which could partly offset the productive advantage of supplementation; this possibility was not quantified here and warrants evaluation in studies incorporating histopathology and energy-balance measurements.

The antioxidant status results clearly demonstrate the efficacy of Nano-ZnO and SEO in mitigating oxidative stress. The significant reduction in serum malondialdehyde (MDA) levels indicates a decrease in lipid peroxidation and oxidative damage [51]. Concurrently, the observed increases in antioxidant enzyme activities, including superoxide dismutase (SOD), glutathione reductase (GR), and total antioxidant capacity (TAC), suggest an enhanced ability to neutralize reactive oxygen species. These effects were most pronounced in the combination group, which is consistent with a combined effect of Nano-ZnO and SEO; because the treatments were compared by one-way ANOVA rather than in a factorial arrangement, no Nano-ZnO × SEO interaction was tested and the responses cannot be attributed to synergy. Zinc is known to induce antioxidant enzyme systems and stabilize cellular membranes, while phytogenic compounds such as those in sesame oil provide additional free radical scavenging activity [52,53]. Together, these mechanisms contribute to improved oxidative balance under heat stress conditions.

The mineral profile is consistent with increased systemic retention and tissue accumulation of zinc in the Nano-ZnO group. Absolute zinc bioavailability was not determined, because no zinc balance or comparative-slope assay was performed, and the present findings therefore indicate greater retention and tissue deposition rather than measured bioavailability. Rabbits supplemented with Nano-ZnO exhibited the highest serum zinc and magnesium concentrations together with the highest hepatic zinc concentration. Hepatic zinc concentrations in the SEO and combination groups were, by contrast, significantly lower than in the control group, so the hepatic response was not a uniform consequence of supplementation and cannot be interpreted as generalized zinc loading. Serum calcium was significantly lower in the Nano-ZnO and combination groups than in the control group, whereas serum phosphorus followed a different pattern, being lowest in the Nano-ZnO group and highest in the combination group. The reduced serum calcium concentration may be associated with antagonism between zinc and calcium at shared intestinal transport pathways, with zinc-related changes in calcium-binding and transport proteins, or with altered renal handling or skeletal partitioning of calcium; the divergent phosphorus response may similarly reflect shifts in the balance between absorption, renal excretion, and tissue deposition [21,27]. None of these mechanisms were directly evaluated here: no calcium or phosphorus balance was measured, no calciotropic hormone such as parathyroid hormone or 1,25-dihydroxyvitamin D was assayed, and no bone mineral measurement was performed. The mineral findings are therefore treatment-related associations of undetermined mechanisms, and the reduced serum calcium concentration should be regarded as an outcome requiring specific investigation rather than as a beneficial effect. These findings indicate that Nano-ZnO supplementation was associated with altered mineral status in growing rabbits.

Regarding serum biochemical parameters, the lack of significant changes in total protein, albumin, cholesterol, and urea suggests that dietary treatments did not adversely affect protein metabolism or general metabolic status. However, the increase in globulin levels and the corresponding reduction in the albumin-to-globulin (A/G) ratio may indicate an altered immunological or humoral status in the supplemented groups [20]. Because no specific immune markers were measured immunoglobulin classes (IgG, IgM, IgA), acute-phase proteins, and lymphocyte subsets were not determined, and serum protein electrophoresis was not performed. The globulin fractions responsible for the increase were not identified and this change cannot be interpreted as improved immune function. Furthermore, the reduction in serum creatinine observed in rabbits receiving SEO may reflect adequate renal clearance and normal protein metabolism under thermal stress rather than a definitive enhancement of baseline renal functional capacity [54]. Serum creatinine is additionally influenced by muscle mass, dietary protein supply, and hydration status, and no glomerular filtration rate estimate, urinalysis, or renal histopathology was obtained; a single lower creatinine value therefore does not establish improved renal function. Overall, the present findings indicate that dietary supplementation with Nano-ZnO and the sesame-derived oil, alone or in combination, was associated with improved growth performance, nutrient digestibility, and antioxidant status in growing rabbits under the heat-stress conditions prevailing during the trial.

The present findings should be interpreted in light of certain methodological limitations. First, the four diets were compared as independent treatments by one-way analysis of variance, and the design was not factorial; consequently, the Nano-ZnO × SEO interaction was not tested, and the responses of the combination group are reported as combined effects and cannot be described as synergistic. Second, a single inclusion level of each additive was evaluated, so the data do not constitute a dose–response study and do not identify an optimal supplementation level. Third, organ health was assessed only through relative organ weights and a limited serum biochemical panel; no histopathological examination and no organ-specific functional assay were performed, so neither an adverse effect nor the absence of one can be established for the liver and kidney. Fourth, zinc status was characterized by serum and hepatic concentrations rather than by a balance or comparative-slope assay, so retention and tissue deposition were measured but bioavailability was not. Fifth, the commercial sesame essential oil (SEO) product was not chemically characterized prior to dietary inclusion; therefore, the concentrations of specific bioactive constituents such as sesamin, sesamol, and sesamolin were not determined. Furthermore, the concentration of SEO-derived bioactive compounds was not verified analytically in the finished pelleted diets, and their stability during feed processing could not be assessed. Because pelleting temperature was not recorded during feed manufacturing and no post-pelleting chemical analyses were performed, the recovery and retention of SEO bioactive compounds in the finished diets remain unknown. Consequently, the observed biological responses can be attributed only to the supplemented SEO product as supplied and not to verified concentrations of individual bioactive components. Sixth, nutrient digestibility was measured only during the final five days of the feeding period and under a 24-h feed-withdrawal protocol, so early-phase responses to supplementation were not captured. Seventh, treatment-specific records of additive cost, feed manufacturing, labour, and other farm inputs were not collected, and no economic or profitability analysis can therefore be derived from these data. Future investigations incorporating chemical profiling (e.g., GC-MS), post-pelleting recovery assays, factorial dose–response studies, histopathology, mineral balance measurements, verified additive concentrations, and full economic recording are required to address these limitations.

## 5. Conclusions

Under severe to very severe heat-stress conditions, dietary supplementation with nano-zinc oxide (60 mg/kg diet), sesame essential oil (300 mg/kg diet), or their combination improved growth performance, feed efficiency, nutrient utilization, and antioxidant status in growing rabbits. Nano-ZnO alone produced the greatest improvement in body weight gain, whereas the combined supplementation yielded the strongest antioxidant responses and highest ether extract digestibility. The present findings indicate that both additives were effective when used individually, and the results do not demonstrate that combined supplementation is required to mitigate heat stress. Because only one inclusion level of each additive was evaluated and treatments were analyzed as independent groups, optimal supplementation levels and potential Nano-ZnO × SEO interactions could not be determined. Additional studies are needed to evaluate the biological significance of the observed changes in mineral metabolism and organ weights and to establish optimal supplementation strategies under commercial production conditions.

## Figures and Tables

**Table 1:** Ingredients and chemical composition of the basal diet fed to growing rabbits.

Ingredients	% Diet	Analyzed Nutrient Composition	%
Yellow corn	32.5	DM %	91.8
Wheat bran	20.5	CP %	16.35
Soybean meal (CP, 44%)	18.3	CF %	12.33
Rice straw	14.0	EE %	3.14
Lucerne hay	5.0	Ash %	9.85
Alfalfa hay	5.0	Calcium %	2.41
Sunflower meal	2.0	Phosphorus %	1.25
Limestone	2.0	Zinc (ppm)	84.81
NaCl	0.3	Gross energy (MJ/Kg)	15.85
Premix * (vitamin-mineral)	0.3		
DL-methionine	0.1		
Total	100		

Values of ingredients are expressed as a percentage (%) of the diet, and nutrient composition is presented on a dry matter basis unless otherwise stated. * Premix supplied the following per kilogram of diet: vitamin A, 10,000 IU; vitamin D_3_, 900 IU; vitamin E, 50 mg; vitamin K, 2 mg; vitamin B_1_, 2 mg; vitamin B_6_, 2 mg; vitamin B_12_, 0.01 mg; niacin, 50 mg; folic acid, 5 mg; pantothenic acid, 20 mg; biotin, 0.2 mg; choline chloride, 1200 mg; Fe, 75 mg; Mn, 8.5 mg; Zn, 70 mg; Cu, 0.1 mg. The analyzed zinc concentration of the unsupplemented basal diet was 84.81 mg Zn/kg diet and represents the combined contribution of the feed ingredients and vitamin–mineral premix. The addition of 60 mg Nano-ZnO/kg diet supplied 48.20 mg elemental Zn/kg diet, calculated based on the molecular proportion of Zn in ZnO (80.34%). Therefore, the calculated total zinc concentrations were 84.81 mg Zn/kg in the control and SEO diets and 133.01 mg Zn/kg in the Nano-ZnO and combination diets. DM, dry matter; CP, crude protein; CF, crude fiber; EE, ether extract.

**Table 2:** Environmental conditions during the experimental period: 10-day period averages ambient temperature (°C), relative humidity %, and temperature–humidity index (THI) of growing rabbits under heat stress.

Items	MIN Temp			MAX Temp		
	Temp (°C)	Hum %	MIN THI	Temp (°C)	Hum %	MAX THI
45–55 d	25	53.71	23.479	33.28	42.85	29.940
55–65 d	25.22	56.25	23.756	34.12	42.12	30.586
65–75 d	24.37	59.71	23.126	33.00	41.14	29.606
75–85 d	23.42	58.57	22.269	33.14	41.00	29.714
85–95 d	25.55	54.71	23.990	33.00	38.85	29.474
95–105 d	24.80	56.73	23.407	33.32	41.35	29.883

Values Represent 10-day period averages of the daily minimum and daily maximum ambient temperature (°C), the relative humidity (%) recorded at those times, and the corresponding minimum and maximum temperature–humidity index (THI). MIN THI and MAX THI are the THI values calculated from the minimum and maximum temperature–humidity pairs, respectively; no separate 24-h mean THI was computed. THI was calculated according to the equation described in Materials and Methods. Heat-stress classifications were applied according to Marai et al. [34].

**Table 3:** Effects of Nano-ZnO and sesame essential oil supplementation on growth performance of growing rabbits under heat stress.

Items	Treatments				SEM	*p*-Value
	Control	Nano-ZnO	SEO	Combination		
Body weight g/rabbit						
IW (45 days)	1132.00	1144.00	1143.50	1131.30	5.079	0.716
65 days	1630.50 ^b^	1723.00 ^a^	1702.00 ^a^	1662.20 ^b^	8.058	0.001
85 days	2180.50 ^c^	2333.50 ^a^	2266.50 ^b^	2237.00 ^b^	12.621	0.001
105 days	2730.50 ^c^	2952.00 ^a^	2832.00 ^b^	2829.40 ^b^	16.203	0.001
Body weight gain g/rabbit						
45–65 d	498.50 ^c^	579.00 ^a^	558.50 ^ab^	530.90 ^bc^	7.678	0.001
65–85 d	550.00	610.50	564.50	574.80	8.753	0.083
85–105 d	550.00 ^c^	618.50 ^a^	565.50 ^bc^	592.40 ^ab^	7.905	0.007
45–105 d	1598.50 ^c^	1808.00 ^a^	1688.50 ^b^	1698.10 ^b^	15.634	0.001
Feed intake g/rabbit						
45–65 d	1640.20 ^b^	1712.50 ^a^	1558.50 ^c^	1624.00 ^bc^	14.811	0.001
65–85 d	1848.60 ^b^	1997.00 ^a^	1854.00 ^b^	1894.50 ^b^	16.637	0.002
85–105 d	2038.60 ^b^	2155.50 ^a^	2054.00 ^b^	2094.50 ^ab^	15.727	0.033
45–105 d	5527.40 ^b^	5865.00 ^a^	5466.50 ^b^	5613.00 ^b^	37.888	0.001
Feed conversion ratio						
45–65 d	3.299 ^a^	2.963 ^bc^	2.799 ^c^	3.072 ^b^	0.041	0.001
65–85 d	3.460	3.247	3.239	3.311	0.052	0.893
85–105 d	3.745	3.504	3.641	3.542	0.050	0.336
45–105 d	3.460 ^a^	3.247 ^b^	3.239 ^b^	3.311 ^b^	0.027	0.009

Treatment means and the pooled standard error of the mean (SEM) are presented in separate columns; the SEM column reports a single pooled SEM per variable and is not an individual standard error attached to each mean. Means within a row with different superscripts (a–c) differ significantly (*p* < 0.05). Control: basal diet without supplementation; Nano-ZnO: basal diet + nano-zinc oxide (60 mg/kg); SEO: basal diet + SEO (300 mg/kg); Combination: basal diet + Nano-ZnO (60 mg/kg) and SEO (300 mg/kg).

**Table 4:** Effects of dietary Nano-ZnO and sesame essential oil on nutrient digestibility of growing rabbits.

Items	Treatments				SEM	*p*-Value
	Control	Nano-ZnO	SEO	Combination		
DM %	67.14 ^b^	69.28 ^ab^	71.57 ^a^	71.90 ^a^	0.593	0.009
EE %	58.71 ^b^	59.36 ^b^	60.79 ^b^	64.94 ^a^	0.525	0.001
CP %	64.59 ^b^	70.21 ^a^	71.06 ^a^	71.54 ^a^	0.587	0.001
CF %	52.07	52.84	53.79	51.84	0.466	0.458

Treatment means and the pooled standard error of the mean (SEM) are presented in separate columns; the SEM column reports a single pooled SEM per variable and is not an individual standard error attached to each mean. Means within a row with different superscripts (a, b) differ significantly (*p* < 0.05). Control: basal diet without supplementation; Nano-ZnO: basal diet + nano-zinc oxide (60 mg/kg); SEO: basal diet + SEO (300 mg/kg); Combination: basal diet + Nano-ZnO (60 mg/kg) and SEO (300 mg/kg). (*n* = 10 per treatment group) DM, dry matter; CP, crude protein; EE, ether extract; CF, crude fiber.

**Table 5:** Effects of Nano-ZnO and sesame essential oil (SEO) supplementation on carcass characteristics of growing rabbits.

Items	Treatments				SEM	*p*-Value
	Control	Nano-ZnO	SEO	Combination		
Dressing %	47.34	49.07	48.53	47.38	0.320	0.142
Liver %	3.139 ^b^	2.882 ^b^	3.082 ^b^	3.609 ^a^	0.063	0.001
Lungs %	0.634	0.657	0.719	0.688	0.015	0.253
Heart %	0.271	0.286	0.297	0.276	0.007	0.652
Head %	5.205	5.161	5.651	5.766	0.098	0.057
Kidney %	0.677 ^b^	0.662 ^b^	0.790 ^ab^	0.840 ^a^	0.024	0.018
Small intestine %	6.978	6.416	7.695	7.399	0.195	0.105
Cecum %	0.538	0.520	0.526	0.637	0.019	0.125

Treatment means and the pooled standard error of the mean (SEM) are presented in separate columns; the SEM column reports a single pooled SEM per variable and is not an individual standard error attached to each mean. Means within a row with different superscripts (a, b) differ significantly (*p* < 0.05). Control: basal diet without supplementation; Nano-ZnO: basal diet + nano-zinc oxide (60 mg/kg); SEO: basal diet + SEO (300 mg/kg); Combination: basal diet + Nano-ZnO (60 mg/kg) and SEO (300 mg/kg).

**Table 6:** Effects of Nano-ZnO and sesame essential oil (SEO) supplementation on serum mineral concentrations in growing rabbits.

Items (ppm)	Treatments				SEM	*p*-Value
	Control	Nano-ZnO	SEO	Combination		
Zn	6.276 ^c^	10.357 ^a^	5.215 ^d^	7.233 ^b^	0.402	0.001
Mg	214.65 ^b^	217.34 ^a^	211.75 ^c^	201.68 ^d^	1.266	0.001
P	940.41 ^b^	867.83 ^c^	940.12 ^b^	1010.33 ^a^	10.703	0.001
Ca	1342.46 ^a^	1161.71 ^c^	1308.21 ^b^	1132.56 ^d^	18.939	0.001

Treatment means and the pooled standard error of the mean (SEM) are presented in separate columns; the SEM column reports a single pooled SEM per variable and is not an individual standard error attached to each mean. Means within a row with different superscripts (a–d) differ significantly (*p* < 0.05). Control: basal diet without supplementation; Nano-ZnO: basal diet + nano-zinc oxide (60 mg/kg); SEO: basal diet + SEO (300 mg/kg); Combination: basal diet + Nano-ZnO (60 mg/kg) and SEO (300 mg/kg).

**Table 7:** Effects of Nano-ZnO and sesame essential oil on serum biochemical parameters of growing rabbits.

Items	Treatments				SEM	*p*-Value
	Control	Nano-ZnO	SEO	Combination		
Total protein (g/dL)	4.800	5.437	5.247	5.255	0.097	0.115
Albumin (g/dL)	2.162	1.973	1.942	2.123	0.046	0.258
Globulin (g/dL)	2.638 ^b^	3.463 ^a^	3.305 ^a^	3.132 ^a^	0.094	0.004
A/G ratio	0.818 ^a^	0.575 ^b^	0.598 ^b^	0.683 ^b^	0.0267	0.001
Total cholesterol (mg/dL)	162.21	175.69	204.18	196.99	7.188	0.139
Creatinine (mg/dL)	2.361 ^a^	2.213 ^a^	1.677 ^b^	2.318 ^a^	0.070	0.001
Urea (mg/dL)	55.819	58.095	52.930	53.549	1.501	0.635

Treatment means and the pooled standard error of the mean (SEM) are presented in separate columns; the SEM column reports a single pooled SEM per variable and is not an individual standard error attached to each mean. Means within a row with different superscripts (a, b) differ significantly (*p* < 0.05). Control: basal diet without supplementation; Nano-ZnO: basal diet + nano-zinc oxide (60 mg/kg); SEO: basal diet + SEO (300 mg/kg); Combination: basal diet + Nano-ZnO (60 mg/kg) and SEO (300 mg/kg).

## Data Availability

The original contributions presented in this study are included in the article. Further inquiries can be directed to the corresponding author.
